# Cardiovascular Health of the Balearic Islands (Spain) After the COVID-19 Pandemic

**DOI:** 10.3390/jcm14020511

**Published:** 2025-01-15

**Authors:** Hugo Del Castillo, José M. Gámez

**Affiliations:** 1 Department of Cardiology, University Hospital Son Llatzer, 07198 Palma de Mallorca, Spain; 2 Department of Medicine, Balearic Islands University, 07122 Palma de Mallorca, Spain; 3 CIBER de Fisiopatología de la Obesidad y la Nutrición (CIBEROBN CB 12/03/30038), Instituto de Salud Carlos III, 28029 Madrid, Spain

**Keywords:** COVID-19, pandemic, cardiovascular health, cardiovascular diseases, Balearic Islands, Spain

## Abstract

**Background/Objectives**: The COVID-19 pandemic caused healthcare managers to reallocate resources from other areas in order to handle the outbreak. Cardiovascular usual care was especially affected during the first wave. **Methods**: We analyzed the short- and mid-term impact of the resource reallocation on cardiovascular health, focusing on the Balearic Islands in Spain. Data from 2019 to 2021 of the regional healthcare system and from the national and regional stats center were collected in order to analyze the variations in type of admissions, main causes of cardiovascular mortality, and the correlation between the monthly variation in those causes of mortality and total cardiovascular mortality. **Results**: The year 2021 showed worse results in terms of healthcare outcomes compared to the pre-pandemic period, with a population-adjusted cardiovascular mortality increase of 4.8% and heart failure and hypertension being the main drivers (6.4% and 26.45%, respectively). However, this impact was not homogeneous in both pandemic years. In 2020, the main driver for the cardiovascular mortality increase was myocardial infarction (R Pearson = 0.655/*p* = 0.021). On the other hand, chronic conditions such as heart failure and hypertension led to the increase in cardiovascular mortality in 2021 (R Pearson = 0.671/*p* = 0.017 and R Pearson = 0.619/*p* = 0.032, respectively). In-hospital resources for COVID-19 showed a positive correlation to cardiovascular mortality in 2021; however, it did not reach statistical significance (R Pearson = 0.41/*p* = 0.1). **Conclusions**: Mid-term cardiovascular health worsened in the Spanish Balearic Island region, with hypertension and heart failure being the main drivers, with important differences in influence between 2020 and 2021. We found a correlation between COVID-19 in-hospital resource allocation and total cardiovascular mortality.

## 1. Introduction

In December 2019, a group of patients in the Hubei province of China, specifically in the city of Wuhan, began to develop pneumonia symptoms with atypical characteristics and a lack of response to conventional treatments. This marked the beginning of the well-known massive spread of SARS-CoV-2 around the world and its devastating consequences, not only on the healthcare front but also economically and socially. Several mechanisms for cardiac damage related to COVID-19 have been proposed. The most popular has been related to the overexpression of angiotensin-converting enzyme 2, which may lead to increased cell permeability able to facilitate penetration of the virus in the myocardium and other tissues [1]. During the first year of the COVID-19 pandemic, health systems had to deal with the growing demand for resources to handle the new situation. This circumstance posed a challenge in trying to maintain the quality of care for other patients admitted for different pathologies. The way to keep quality of care in similar situations has been discussed in previous articles [2]; however, the characteristics of the new outbreak were especially challenging in this regard.

Analyzing changes in the distribution of admissions or emergency visits for certain pathologies can provide further information about the indirect impact of the pandemic on various health systems. A common fact observed in different locations is the decrease in the number of emergency consultations for non-COVID-19-related pathologies. For example, according to the U.S. veterans’ registry, non-COVID emergency admissions fell by 41% during the first four months of the pandemic compared to similar periods of the previous year. In Germany, there was a 30% drop between February and April 2020, and there was a 71% decline in Switzerland compared to pre-pandemic periods [3]. The impact was also felt in other sectors, such as primary care in a European setting. In this case, there was a significant decrease in activity during the first wave, not only because of patients’ fear of consulting but also due to the cancellation of visits by healthcare professionals. However, in primary care, activity recovered during the second wave, reaching pre-pandemic levels. In other parts of the world, such as New Zealand, the decrease in healthcare for non-COVID patients resulted in negative consequences in disease screening and for chronic patients [4].

In 2023, Gajewski and collaborators [5] conducted an observational, multicentric, and multinational study comparing emergency visits during a 3-month period in 2020 with a similar period in 2019. They analyzed a total of 8778 patients, 4447 in 2019 and 4331 in 2020. The results showed significant differences in the distribution of cardiovascular emergencies between the two years studied. The frequency of pulmonary embolism increased in 2020 compared to the previous year, but other cardiovascular diseases declined, notably heart failure, which showed a reduction of more than 50%. Regarding acute coronary syndrome, 2020 showed an increase in ST-elevation myocardial infarction. Meanwhile, De Filippo and colleagues [6] published a retrospective study analyzing admissions of patients with acute coronary syndrome during the first wave in hospitals in northern Italy, all of which were referral centers with primary angioplasty. They also compared the hospitalization rates of the first wave with two pre-pandemic control periods. Of the 547 patients analyzed during the study period, 248 had ST-elevation myocardial infarction. The average daily admissions were 13.3, significantly lower than both control periods (18/day). Additionally, the authors discussed an increase in mortality during this period in Italy that cannot be fully explained by COVID-19. These data are supported by another Italian group, in this case from a region in the south of the country, which reports an overall reduction in cardiovascular admissions during the first wave of the pandemic, with fewer heart attacks, both with and without ST elevation, and fewer admissions for heart failure [7].

Regarding cardiac surgery, a systematic review of articles between January 2019 and August 2022 was published to determine the impact of the reallocation of healthcare resources during the pandemic and how cardiac surgery programs were affected. They found that resources indeed shifted from cardiac surgery programs to other sectors to fight the pandemic. This particularly affected elective surgeries, increasing the rates of urgent surgeries with a consequent rise in mortality and complications [8].

Special attention is also given to admissions for bradyarrhythmias requiring pacemaker implantation. Dell’Era and collaborators [9] published a study reflecting the impact of lockdown and the pandemic period on patients who were candidates for devices to treat bradyarrhythmias. They evidenced a decrease of approximately one urgent pacemaker implantation per day during the pandemic compared to control periods, with the strictest lockdown period being the most affected. They also analyzed how rural areas were affected compared to urban areas, with the former experiencing a greater decline. The drop in scheduled implants was even more pronounced, reducing to almost half. Another study [10] reported a nearly 24% decrease in pacemaker implantations during the confinement months, followed by a subsequent rebound of 107%. Patients requiring urgent pacemakers during the confinement period more frequently presented with hypotension, shock, and the need for temporary pacemakers. In their conclusions, the researchers emphasize the need for bradyarrhythmia patients to seek medical care regardless of the circumstances, as their risk of complications in the absence of proper treatment is very high. Regarding the implantation of defibrillators, Zelijkovic’s group [11] did not find differences in the number of defibrillators implanted during 2020 compared to the control period, while a 45% decrease in pacemaker implantation was observed. However, when analyzing the defibrillators implanted during the confinement, a reduction is observed compared to similar months in the control period, resulting in a ’compensation effect’ in the following months. In relation to the previously mentioned delay in the implantation of defibrillators, a significant increase in out-of-hospital cardiac arrests was documented in the United Kingdom. Experts attribute some of these events to the alteration of the usual dynamics in defibrillator implantation during the COVID-19 pandemic [12]. Focusing on the entire Spanish territory, the defibrillator registry in 2020 revealed a decrease in the implantation of these devices compared to previous years [13].

In short, the emergence of COVID-19 led to a redistribution and reorganization of healthcare systems in providing care to cardiovascular patients. This relocation of resources and its impact on quality of care and medium-term effects on population cardiovascular health is the aim of our study in our area, the Balearic Islands region in Spain, with a population of 1.2 million inhabitants.

## 2. Materials and Methods

### 2.1. Study Design and Population

To carry out the study, data were obtained from public administrative databases belonging to the local health service and the local statistics service, which is a subsidiary of the National Statistics Institute. The data obtained correspond to the years 2019 (pre-pandemic period), 2020 (early pandemic period), and 2021 (late pandemic and post-pandemic period).

Regarding cardiovascular health data, monthly global cardiovascular mortality data were obtained from the Balearic Islands population, as well as divided into the various causes of mortality coded in the official coding manual used in our healthcare system (International Classification of Diseases and Related Health Problems), with a special focus on hypertensive diseases, myocardial infarction, heart failure, and cerebrovascular diseases. For these entities, a correlation analysis for month-by-month variability was performed for the entire studied period and for each of the three years individually, aiming to assess if the resource redistribution during the pandemic changed the correlation pattern between total cardiovascular mortality and each of these entities.

Additionally, data were obtained for outpatient activity, coronary intervention, pacemaker novel implantation, and ICD generator implantation from cardiology services throughout the region. Moreover, data for the number of hospitalizations due to cardiovascular diseases during 2019–2021 were also collected and classified into urgent and scheduled.

Lastly, a specific analysis of the two pandemic years (2020–2021) was conducted, obtaining data on healthcare pressure in terms of the number of stays and in-hospital days for all cardiovascular diseases, hypertensive diseases, myocardial infarction, heart failure, cerebrovascular disease, and COVID-19. The goal of this final section of the study was to assess how the need for hospital resources to care for COVID-19 patients correlated with in-hospital resources in terms of the number of admissions and in-hospital days for cardiovascular diseases.

### 2.2. Statistical Analysis

Initially, a normality and descriptive study was conducted for each variable. For variables with a sample size of less than 50, the Shapiro–Wilk test was used. For variables greater than 50, the Kolmogorov–Smirnov test was used. Based on these tests, the samples were divided into parametric and non-parametric. For correlation tests, Pearson’s R was used for parametric distributions and Spearman’s Rho for non-parametric distributions. A statistical significance value of *p* < 0.05 was established.

## 3. Results

### 3.1. Clinical and Interventional Activity in Cardiology

The activity in cardiology services, both at an outpatient level and an interventional level, decreased in 2021 compared to 2019, especially in outpatient activity in external consultations and percutaneous coronary intervention. However, percutaneous intervention in acute patients, those related to myocardial infarction, remained at similar levels throughout the studied period. In terms of patients presenting with cardiogenic shock associated with cardiac arrest, 2019 was the year with the most admissions (15), followed by 2020 (6), with 2021 being the year with the least codified admissions for cardiac arrest and cardiogenic shock. The greatest drop in outpatient activity occurred in 2020, without fully recovering in 2021. On the other hand, no decrease was observed in the implantation of intracardiac devices, both novel pacemaker implants and defibrillators. These results are shown in detail in Table 1.

**Figure 1:**
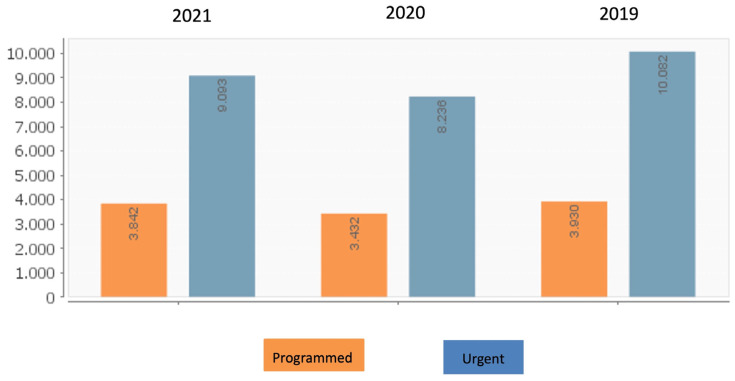
Urgent and regular cardiovascular admissions from 2019 to 2021.

The COVID-19 pandemic led to a sharp reduction in cardiovascular admissions in 2020 compared to 2019, both in urgent and scheduled admissions, without recovering to pre-pandemic levels in 2021. Detailed results are shown in Figure 1 and Supplementary Material. Notice the decrease in both types of admissions in 2020 without a total recovery in 2021.

### 3.2. Cardiovascular Mortality

Regarding total cardiovascular mortality and its main causes, it is observed that, in general terms, there is a widespread increase over the three years studied, except for cerebrovascular disease, which shows a decrease in mortality adjusted per 100,000 inhabitants.

In our study, the conditions that showed the greatest increase in mortality per 100,000 inhabitants in 2021 compared to 2019 are hypertensive diseases and heart failure. Although there is also an increase in mortality due to myocardial infarction, it is less pronounced than in the other conditions. These results are shown in more detail in Table 2 and Supplementary Material.

### 3.3. Main Drivers in Cardiovascular Mortality Increase

In our study, the overall cardiovascular mortality and the main causes of cardiovascular mortality were analyzed month by month during the years 2019–2021, whose descriptive parameters are shown in Table 3 and Supplementary Material.

The correlation data of the different causes of cardiovascular mortality with total cardiovascular mortality vary during the pandemic. The year 2019 shows a predictable pattern, where the main causes of cardiovascular mortality correlate positively month by month with total cardiovascular mortality, with three of these correlations being statistically significant. In 2020, the pattern changes compared to 2019, finding only a significant positive correlation with myocardial infarction. In 2021, the correlation pattern changes again month by month, finding only a statistically significant positive correlation between hypertension and heart failure with total cardiovascular mortality. The detailed correlation results are shown in Table 3.

During the pandemic, there was a diversion of resources from cardiovascular services to care for COVID-19 patients. In 2020, a neutral correlation was observed between hospital care pressure month by month, in the form of the number of stays and the total number of days of hospital stays dedicated to cardiovascular patients and COVID-19 patients. In 2021, hospital resources used for COVID-19 patients were negatively correlated month by month with those used for cardiovascular patients. The detailed correlation results are shown in Table 4.

## 4. Discussion

In our study, hypertensive diseases show a significantly higher mortality rate in 2021 compared to 2019, followed by heart failure, with a weaker variability in ischemic heart disease and an improvement in cerebrovascular disease (mortality adjusted per 100,000 inhabitants). This leads us to think that the effect of COVID-19 may have impaired the quality of care in the management of hypertension and heart failure, two chronic conditions that benefit from continued attention, which was interrupted by the need to care for COVID-19 patients.

A striking finding from part of the study is that, despite the number of admissions prompted by COVID-19, the year in the 2019–2021 series with the highest number of admissions is 2019, both overall and exclusively for cardiovascular diseases. This is mainly due to emergency admissions, which fell more than scheduled admissions and did not recover to the pre-pandemic levels in 2021. This phenomenon is interesting as it suggests that many cardiovascular and other patients stopped consulting hospital emergency services, with a trend that continues beyond the first wave and also leaves its footprint on the days of stay.

Despite the outbreak of COVID-19, with all the days of stays generated, the number of days of stay decreased in 2020 and did not recover to pre-2020 levels in 2021. This suggests that it is not due to the capacity of the healthcare system since the overall use of hospital resources in terms of days of stay is lower during the pandemic than before. However, it might be explained by a paradigm shift in the way outpatient doctors referred to the emergency department, emergency services organization, and patients themselves, creating a different “culture” of emergency service use and greater use of telemedicine.

Another aspect to be discussed is the decrease in the number of admissions for cardiac arrest associated with cardiogenic shock in patients without COVID-19 (usual cardiogenic shock patients). We wanted to analyze if the collateral effects of the pandemic led to a significant change in the number of cardiogenic shocks due to the severity of the cardiovascular disease itself. We expected an increase in this type of admission in 2020 or 2021 compared to the pre-pandemic period, as it could be assumed that usual cardiological patients may seek medical care later or receive impaired quality of care due to the pandemic environment. However, this does not occur in our cohort. It is important to remark that we worked with administrative data based on coding, and we only have cardiogenic shock data from patients managed in cardiology or cardiac surgery services, which is a limitation. Therefore, these data must be interpreted with care.

As has been discussed before, non-COVID-19 patients suffered less cardiogenic shock associated with cardiac arrest episodes (considering the mentioned limitations). Nevertheless, it would be interesting to analyze cardiogenic shock in COVID-19 patients, as it is known that the virus can impact cardiovascular conditions and can also be the cause of myocarditis, which can be a possible cause of cardiogenic shock. During the pandemic, cardiologists and caretakers needed to pay even more attention to the presence of myocarditis by carefully analyzing ECG, echocardiogram, and cardiac magnetic resonance findings [14,15,16].

Heart failure and hypertension were the diseases with the worst increase in mortality rates post-pandemic. Although a direct relationship cannot be inferred, it could be assumed that the need for fewer hospital beds may be linked to fewer resources being allocated or more neglect of these chronic patients who sought medical care. As it is known, hypertension is a chronic disease that benefits from effective outpatient follow-up and is susceptible to acute decompensations [17]. Additionally, it can affect multi-pathological patients who are more vulnerable to long stays. On the other hand, these data are consistent with mortality data from hypertensive diseases, which is the cardiovascular disease whose mortality has worsened the most after the pandemic compared to 2019 [18]. Moreover, the significant worsening of patients with hypertension is notable when the accumulated prevalence of this disease was on a downward trend in the years prior to the pandemic [19].

If attention is paid to resources allocated to acute conditions, acute care for myocardial infarction is hardly affected, with similar behavior in terms of novel pacemaker implants. This may be related to the fact that an acute worsening of healthcare status may be more amenable to being consulted in an emergency department.

The year 2020, corresponding to the first wave and the major shutdown of activity, has a different behavior compared to the following year or late pandemic period (2021) in terms of the relationship between cardiovascular mortality evolution month by month and hypertensive diseases, with no clear correlation between overall cardiovascular mortality and mortality from hypertensive diseases. This may be explained by the poor control that may have been carried out in 2020, which could have had a negative impact on adverse events in 2021, where the correlation is strong. On the other hand, the fact that there were fewer hospitalizations in 2020 could have contributed to worse death coding, as continued hospital care until death allows for more information in terms of coding than a death recorded at home. Following the special behavior of 2020, we see a strong positive correlation, as expected, between mortality from myocardial infarction and cardiovascular mortality; however, this trend did not match when hypertension was analyzed. It is important to note that care for myocardial infarction was barely affected in 2020, and as mentioned before, acute symptoms like those of myocardial infarction could lead to less fear of contagion when seeking medical attention, compared to elevated blood pressure, which often presents no symptoms. In fact, the myocardial infarction-related parameters show a significant correlation with total cardiovascular mortality.

The year 2020 is also special for cerebrovascular disease, which has been declining over the years in terms of mortality adjusted for population. The widespread introduction of direct anticoagulants during the pandemic may have contributed to a decrease [20] in the total cerebrovascular mortality/100,000 inhabitants rate, with this being the only cardiovascular entity showing this downward trend. This fact is particularly noteworthy, considering the pro-thrombotic effect of the COVID-19 infection itself, which could have been expected to result in a higher rate of adverse cardiovascular events in conditions like ischemic stroke associated with thrombosis or embolism. Patients with COVID-19-associated strokes have special characteristics that differentiate them from others, sometimes showing high virulence [21]. The use of direct anticoagulants (which were restricted in the Spanish national health system at the time) to avoid excessive visits to the healthcare system was seen as a useful alternative to vitamin K antagonists in the prevention of ischemic stroke associated with atrial fibrillation, with anticoagulants demonstrating a more favorable risk–benefit profile compared to vitamin K antagonists [22]. This series of advantages at the care level led to an increase in the use of direct anticoagulants nationwide [23]. Considering the unexpected decrease in cerebrovascular disease mortality (caused by both thrombotic and hemorrhagic phenomena) in our study between 2019 and 2021, it could be hypothesized that the increase in the use of direct anticoagulants might have partially contributed to these results, although specific studies are needed to confirm this hypothesis. In Spain, it has been observed that the use of direct anticoagulants during the pandemic led to improved healthcare quality [24].

Regarding heart failure, a correlation is observed between mortality from this entity and total cardiovascular mortality, which is expected and is also statistically significant. This coincides, on the other hand, with an increase in total mortality from heart failure during the pandemic years, which can be explained by a potential loss of quality of care for a disease that requires continuous care. Heart failure and hypertension often occur together; therefore, a similar behavior was found as expected.

It has been proposed that one short-term contributing factor to the increase in adverse events is the reallocation of health resources from other pathologies to COVID-19 patients [24]. In our study, the capacity of the pandemic to impact cardiovascular admissions is intuited, as has already been documented in [25]. Indeed, there is a strong negative correlation between the number of stays for cardiovascular diseases and the number of stays for COVID-19, which can be useful as a reference to indicate that hospital resources may have been diverted from one pathology to another. In fact, in 2021, when the most healthcare resources were needed to care for COVID-19 patients, the least hospital resources in the form of days of stay were used for cardiovascular patients, this negative correlation being significantly stronger than the year of the first wave. In previous paragraphs, some of the differences in the behavior of the different clinical and care pressure parameters analyzed and their relationship with cardiovascular mortality have already been discussed.

Let us now analyze the differences between both years in more detail. One of the main differences comes from the entity that has worsened its data the most after the pandemic, which is hypertensive diseases. In 2020, there is no correlation between mortality from hypertensive diseases and monthly variation in total cardiovascular mortality. However, the influence on total cardiovascular mortality increase in 2021 is much greater and statistically significant. The possibility of certain coding difficulties has already been discussed, but the cumulative effect of a year with a deficit in the quality of care for hypertensive patients may be a more plausible explanation for the poor data for 2021 in terms of total and population-adjusted mortality rates. Heart failure and hypertension often occur together, and this is the case in 2021, when both conditions show a similar correlation pattern with cardiovascular mortality rates. In 2020, there is also a correlation between cardiovascular mortality and mortality due to heart failure (not statistically significant), but the pattern is not the same as arterial hypertension. In 2020, the cause that has the greatest influence on the month-to-month variation in total cardiovascular mortality is myocardial infarction, while in 2021, it is hypertension and heart failure, which are factors with the capacity to increase in-hospital morbidity in patients with COVID-19 [25]. This fact can be explained by the worsening of chronic processes [26], with their complications due to poorer management occurring after one year. Myocardial infarction, a more acute process, presents a more linear behavior, perhaps, as has been commented, because of its acute nature [27].

In 2020, there was a drop in the number of coronary angiograms not related to acute myocardial infarction, both with and without intervention. It might be thought that the fact of revascularizing less could lead to an increase in future mortality due to infarction; however, in our study, this concept is not clear, presenting population-adjusted mortality related to MI with higher values in 2021 than pre-pandemic, but in a less marked way compared to other chronic pathologies mentioned. In this case, the debate could be opened about the suitability of medical treatment compared to interventionism in chronic coronary syndromes, as various authors have suggested [28]. However, from our study, it cannot be inferred that the reduction in coronary interventionism not related to infarction has been directly replaced by medical treatment. It would have been interesting to analyze the time from symptom to onset to percutaneous intervention, how antithrombotic therapy was carried out in the different periods analyzed, and to study in-depth thrombotic and bleeding complications. However, the use of administrative databases instead of clinical ones served as a limitation and can be the target of further studies in our setting.

In our study, 2021 surpasses 2020 in terms of the number of stays and the number of beds, and this is strongly and statistically significantly related in 2021 to hospital resources allocated in the form of beds and days of stay to cardiovascular patients. Therefore, it could be hypothesized that, in 2021, the “perfect storm” occurs, combining the loss of quality of care that began in 2020 and a greater need for cardiovascular beds, as well as a diversion of hospital resources to COVID-19 patients as a result of a sharp increase in care needs to treat COVID-19. According to the data analyzed, 2021 had the worst results for cardiovascular health. We found a positive correlation between in-hospital resources for COVID-19 and total cardiovascular mortality, especially in 2021; however, this finding did not reach statistical significance.

Finally, the incidence and mortality of COVID-19 in the Balearic Islands is relatively low, and there are no deaths codified related to multisystemic inflammatory syndrome due to COVID-19 [29] compared to other regions (probably favored by the fact that it is an island cluster region), so it does not seem very likely that the entire increase in cardiovascular mortality shown in the results section can be attributed to cardiovascular decompensations due to COVID-19 as the main cause. It could have a greater indirect relationship with the COVID-19 disease in the form of diversion of resources, lack of seeking medical assistance due to fear of contagion, healthcare overload, closure of activity, etc. [30].

The main limitation of our study is inherent to the use of administrative databases. For this reason, it has not been possible to analyze some relevant variables in this study, such as the implementation of specific therapeutic measures or adherence to clinical practice guidelines or more specific economic data, the availability of which could have influenced the results. On the other hand, since we work with anonymous patient data, long-term follow-up of the patients analyzed is not possible.

It would also be interesting to develop a future study targeting the impact of vaccination on cardiovascular patients in our setting, as the vaccines were one of the cornerstones for fighting COVID-19 [31].

Furthermore, the absence of data on outpatient activity in primary care and other services involved in the care of cardiovascular patients limited our analysis to hospital data and outpatient activity in cardiology services.

## 5. Conclusions

The COVID-19 pandemic led to a reduction in outpatient activity in the cardiology services of the Balearic Islands in hospital and outpatient settings. However, the care of acute myocardial infarction was barely affected in terms of coronary intervention procedures. The more hospital resources were allocated to COVID-19, the less were allocated to cardiovascular diseases.

Cardiovascular mortality increased in the Balearic Islands in 2021 compared to 2019, with acute conditions being more important in 2020 and chronic diseases in 2021. In 2021, the most hospital resources during the pandemic were allocated to treating COVID-19, and the year had the worst cardiovascular health data. Even though the data did not reach statistical significance, our study shows a trend for worse cardiovascular outcomes when more resources were allocated to COVID-19 patients.

## Figures and Tables

**Table 1:** Activity in cardiology services throughout the Balearic Islands.

	2019	2020	2021	Difference 2021–2019 (%)
Coronary intervention	1272	1249	1164	−8.6
Coronary intervention related to MI	704	702	697	−1
Coronary intervention without MI	568	547	467	−17.8
Pacemaker implantation (first implant)	301	307	365	21.2
ICD generators (first implant and replacement)/1,000,000 inhabitants	121	122	119	−1.7
Outpatient clinic visits	114,208	96,005	109,732	−4

MI: myocardial infarction; ICD: implanted cardiac defibrillator.

**Table 2:** Mortality rates between 2019 and 2021.

	2019	2020	2021	Difference 2019–2021 (%)
Mortality/100,000				
HF	31.47	31.85	33.62	6.4%
IC	46.32	41.15	47.73	2.96%
HT	24.37	29.96	33.13	26.45%
CVD	36.98	34.65	34.77	−6%
TCarVD	194.7	195.25	204.66	4.8%
Total mortality rates				
TCarVD	2202	2252	2405	8.44
HT	292	364	404	27.72
MI	206	190	194	−6.18
HF	377	387	410	8.048
CVD	443	421	424	−4.48

Comparison of cardiovascular mortality between 2019 and 2021. HF: heart failure; IC: ischemic cardiomyopathy; HT: hypertension; CVD: cerebrovascular disease; TCarVD: total cardiovascular disease.

**Table 3:** Correlation between main causes of cardiovascular mortality and total cardiovascular mortality from the pre-pandemic period (2019–2021).

Correlation	R Pearson	*p* Value
2019		
TCarVD-HT M	0.712	0.009
TCarVD-MI M	0.620	0.032
TCarVD-HF M	0.837	0.001
TCarVD-CVD M	0.5	0.083
2020		
TCarVD-HT M	0.005	0.098
TCarVD-MI M	0.655	0.021
TCarVD-HF M	0.357	0.254
TCarVD-CVD M	0.150	0.643
2021		
TCarVD-HT M	0.671	0.017
TCarVD-MI M	0.451	0.141
TCarVD-HF M	0.619	0.032
TCarVD-CVD M	0.134	0.678

Notice that the pattern of correlation changed after the outbreak. M: mortality; HF: heart failure; HT: hypertension; TCarVD: total cardiovascular disease, MI: myocardial infarction, CVD: cerebrovascular disease.

**Table 4:** Correlation between in-hospital resources needed for COVID-19 and cardiovascular parameters in 2020 and 2021.

Correlation	R Pearson	*p* Value
2020		
NoS COVID-NoS TCarVD	0.2	0.55
DoS COVID-DoS TCarVD	0.09	0.832
TCarVDM-DoS COVID	0.22	0.106
TCarVDM-NoS COVID	0.102	0.081
2021		
NoS COVID-NoS TCarVD	−0.692 *	0.013
DoS COVID-DoS TCarVD	−0.610	0.035
TCarVDM-DoS COVID	0.352	0.200
TCarVDM-NoS COVID	0.412	0.100

Cardiovascular parameters (cardiovascular mortality and hospital resources needed for cardiovascular patients). TCarVD (total cardiovascular diseases), NoS COVID (total number of stays for COVID-19); DoS COVID (total days of stay for COVID-19); NoS TCarVD (total number of stays for cardiovascular diseases); DoS TCarVD (total days of stay for cardiovascular diseases); TCarVDM (total cardiovascular mortality). * In this case, Spearman’s Rho, which was used as the variable, was non-parametric.

## Data Availability

Data is contained within the article or Supplementary Materials. The datasets used and/or analyzed during the current study are available from the corresponding author upon reasonable request.

## Supplementary Materials

The following supporting information can be downloaded at: https://www.mdpi.com/article/10.3390/jcm14020511/s1, Table S1: COVID-19 Deaths in Spain; Table S2: Cardiovascular Admissions; Table S3: Cardiovascular Mortality and its Main Causes in 2019; Table S4: Stays for COVID-19 from the Years 2020 and 2021; Table S5: Descriptive Analysis from 2020; Table S6: Descriptive Analysis from 2021.
