# Supplementary material for: Cardiovascular Health of the Balearic Islands (Spain) After the COVID-19 Pandemic
Source: J Clin Med. 2025 Jan 15;14(2):511. doi: 10.3390/jcm14020511 (PMC11766286; doi:10.3390/jcm14020511)
Supplement: Supplementary file 1 [file jcm-14-00511-s001.zip › jcm-3404975-supplementary.pdf]

Table S1. COVID deaths in Spain

Year	Month	Total COVID deaths
**2020**	March	60
**2020**	April	144
**2020**	May	22
**2020**	June	1
**2020**	July	3
**2020**	August	33
**2020**	September	88
**2020**	October	46
**2020**	November	56
**2020**	December	92
**2021**	January	167
**2021**	February	100
**2021**	March	19
**2021**	April	10
**2021**	May	7
**2021**	June	3
**2021**	July	30
**2021**	August	89
**2021**	September	34
**2021**	October	15
**2021**	November	16
**2021**	December	65

Total COVID deaths in the Balearic Islands Region in Spain between March 2020 and December 2021

Table S2. Cardiovascular Admissions

Year	2019	2020	2021
Urgent	3930	3432	3842
Programmed	10082	8236	9093

Urgent and programmed cardiovascular admissions from 2019 to 2021

Table S3. Cardiovascular Mortality and its Main Causes in 2019

2019	N months	Min	Max	Mean	SD
**M TCarVD**	12	151.00	246.00	183.50	31.41
**M HT**	12	16.00	32.00	24.33	5.61
**M MI**	12	10.00	25.00	17.16	4.66
**M HF**	12	21.00	53.00	31.41	8.69
**M CVD**	12	30.00	45.00	36.91	5.08

Descritptive analysis of total cardiovascular mortality and main causes of cardiovascular mortality in 2019. HF: Heart failure; MI: Myocardial infarction; HT: Hyperthension; CVD: Cerebrovascular disease; TCarVD: Total cardiovascular disease; N: number; SD: standard deviation

Table S4. Stays for COVID-19 from years 2020 and 2021

COVID-19	NoS 2020	DoS 2020	MoS2020	NoS2021	NoS2021	MoS2021
**Total**	2440	28893	11.8	4557	65149	14.3
**Men**	1453	17941	12.3	2601	38143	14.7
**Women**	987	10953	11.1	1957	27007	13.8

NoS: number of stays; DoS: total days of stay; MoS: mean of stay.

Table S5. Descriptive Analysis from 2020

2020	N months	Min	Max	Mean	SD
**M TCarVD**	12	138.00	233.00	187.6667	27.53950
**M HT**	12	16.00	32.00	24.3333	5.61384
**M MI**	12	10.00	25.00	17.1667	4.66775
**M HF**	12	21.00	53.00	31.4167	8.99958
**M CVD**	12	30.00	45.00	36.9167	5.08935
**NoS TCarVD**	12	586.00	1181.00	972.2500	158.89970
**DoS TCarVD**	12	6149.00	11682.00	8991.1667	1385.55173
**MoS TCarVD**	12	8.30	12.20	9.3417	1.18280
**NoS HT**	12	121.00	219.00	171.2500	30.52607
**DoS HT**	12	958.00	1786.00	1340.9167	285.04560
**NoS MI**	12	71.00	128.00	107.2500	16.32135
**DoS MI**	12	470.00	996.00	730.5000	156.10049
**NoE HF**	12	42.00	92.00	66.7500	16.27114

**DoS HF**	12	456.00	1266.00	859.0833	306.30598
**NoE CVD**	12	136.00	241.00	185.5833	29.21848
**DoE CVD**	12	2255.00	5072.00	3250.6667	781.79448

Descriptive analysis 2020 corresponding to the first COVID-19 year. M: mortality; Mos: mean of Stage; NoE: number of stages; DoS: days of stage; HF: heart failure; IC: ischaemic cardiomyopathy (IC); HT: hypertension; CVD: cerebrovascular disease; TCarVD: total cardiovascular disease; SD: standard deviation.

Table S6. Descriptive Analysis from 2021

**2021**	**N months**	**Min**	**Max**	**Mean**	**SD**
**M TCarVD**	12	173	268	200.4167	31.41933
**M HT**	12	26	36	30.3333	4.03019
**M MI**	12	7	21	15.8333	4.48904
**M HF**	12	25	40	32.25	5.64277
**M CVD**	12	19	44	35.0833	7.63316
**MoSTCardVD**	12	8.2	9,1	8.7	0.31042
**NoE HT**	12	120	206	170.75	26.39947
**DoSHT**	12	1133	1781	1401	201.90457
**NoE MI**	12	99	143	123.1667	12.26105
**DoE MI**	12	717	1160	910.0833	155.26192
**NoE HF**	12	61	123	92.3333	19.78674
**DoE HF**	12	463	1316	897.8333	249.91083
**NoE CVD**	12	173	245	203.4167	24.07737
**DoE CVD**	12	2016	3831	3175.0833	534.85877
**NoS TCarVD**	12	919	1805	1137.90	232.164
**Dos TCarVd**	12	7951	11225	9390.90	950.14

Descriptive analysis 2021 corresponding to the second COVID-19 year. M: mortality; Mos: mean of Stage; NoE: number of stages; DoS: days of stage; HF: heart failure; IC: ischaemic cardiomyopathy (IC); HT: hypertension; CVD: cerebrovascular disease; TCarVD: total cardiovascular disease; SD: standard deviation.
